# Ad hoc and Fast Forward: The Science of Hormesis Growth and Development

**DOI:** 10.1289/ehp.0900761

**Published:** 2009-05-20

**Authors:** Paul Mushak

**Affiliations:** PB Associates, Durham, North Carolina USA

**Keywords:** hormesis modeling, risk assessment, risk characterization, regulatory policy

## Abstract

**Background:**

Hormesis is a binary response phenomenon with low-dose stimulation (or inhibition) of effects by substances producing opposite high-dose responses. Hormesis, after decades of obscurity, has undergone a renaissance in recent years, with rapid growth benefiting greatly from the systematized efforts of such proponents as the hormesis group at the University of Massachusetts-Amherst led by Edward J. Calabrese.

**Objective:**

In this commentary I analyze chemical hormesis methodology with reference to ad hoc scientific approaches for defining and characterizing hormesis.

**Discussions:**

Proponents of hormesis have attempted a scientific characterization of hormesis through a battery of ad hoc methodologies using unvalidated criteria and other mechanisms for persistent database searches rather than through *de novo* hypothesis testing specific for hormesis. Here I discuss various scientific problems with this search-over-experiment approach, as well as other aspects of attempts at defining and characterizing the field.

**Conclusions:**

Wide acceptance of hormesis by the broad scientific community and adoption of hormesis by public agencies for inclusion in health and regulatory policies have not occurred. Reasons may include the singular nature of hormesis research and directions followed in hormesis methodologies.

Hormesis can be broadly defined as a binary response phenomenon with low-dose stimulation (or inhibition) of effects by substances producing opposite responses at a high dose. A more far-ranging definition from Calabrese and Baldwin (2002) states hormesis to be an adaptive response having similar biphasic dose–response features as to amplitude and range, with the stimulatory response being either directly induced or compensatory following disturbance of homeostasis. This definition was expanded by Calabrese et al. (2007a), proposing to stratify hormesis into three categories of hormetic expressions to better accommodate varying perceptions of the term.

Work by hormesis proponents over the last 10–15 years has included developing a body of ad hoc scientific approaches specific to hormesis and mixing ad hoc science with relatively strong advocacy, using diverse mechanisms for hormesis development as a biologic topic in dose–response toxicology, human health risk assessment, and environmental regulatory policies.

The development and extensive employment of ad hoc methodologies for hormesis characterization and presenting that characterization within an idiosyncratic, tailored vocabulary are discussed below.

Most of the systematized scientific activity on hormesis, organized to rationalize and characterize the topic *per se*, has been done by the University of Massachusetts-Amherst (UMass-Amherst) hormesis program led by Edward J. Calabrese. The bulk of that activity addressed chemical hormesis, with radiation hormesis getting relatively less attention.

## Ad hoc Scientific Methodologies for Detecting and Characterizing Hormetic Phenomena

Promoting hormesis as a subject for study and characterization has followed various rationales and methodologies and employed ad hoc pathways, that is, methods specific to the case of hormesis characterization. These largely originated with Calabrese and colleagues. These workers did not address the nature and scope of putative hormetic phenomena by doing *de novo* hypothesis testing using only validly designed and executed studies for hormesis characterization. They elected to establish a searching and screening methodology for hormesis commonality in the published literature using ad hoc identification and characterization criteria. Calabrese (2001) rationalized this choice on the basis of the relatively high cost and difficulty of carrying out *de novo* experimental studies when meaningful outcomes were far from assured.

This search-over-experiment strategy, however, requires fundamental assumptions about the required ad hoc battery of *de novo* search and assessment criteria, the relative complexity and validity of which determine the extent to which claims for hormesis should be judged by the scientific community. They include the following: *a*) quantitative characteristics of hormesis in selected journals approximate those in all publications or in nature; *b*) the statistical complexities and limitations underlying data searching for hormetic phenomena do not void the validity of the results; *c*) any ad hoc search criteria evolved for hormesis searches have been validated for reliability in terms of sensitivity and specificity; and *d*) search methodologies and their results are fully transparent to outside parties.

The UMass-Amherst–based articles, summaries, and commentaries published mainly in the last 8–10 years deal with discrete but expanding blocks of data, some of which have been presented in the open literature in confusing and overlapping time frames (e.g., Calabrese 2008; Calabrese and Blain 2005).

Two core claims over the past 8–10 years—that hormesis is a common biological phenomenon and that it is relatively more common in terms of dose–response models than threshold responses—are principally rooted in publications winnowed from a large data set of articles noted below. The first claim, of significant frequency, was by Calabrese and Baldwin (2001). These authors claimed primacy of hormesis over threshold dose responses in 2003 (Calabrese and Baldwin 2003b). Published materials by Calabrese and colleagues on either side of this time frame consist largely of papers leading up to this key pair of articles or are subsequent additions of papers from other databases, such as those data for substances examined within the National Toxicology Program (NTP) (Calabrese and Baldwin 2003a) or by the National Cancer Institute (NCI) (Calabrese et al. 2006, 2007b, 2008; Crump 2007).

None of the key databases evaluated by the UMass-Amherst hormesis group and noted here (Calabrese and Baldwin 2001, 2003a, 2003b; Calabrese et al. 2006, 2008) were developed to demonstrate or characterize hormesis. Hormesis-like data were sought in databases created for other purposes but having doses judged to be below a no observed adverse effect level (NOAEL). Screening for hormetic phenomena through retrospective and ad hoc quantification criteria in studies not designed to show hormetic phenomena creates various problems. These stratify into problems with initial selection of databases and problems internal to processing these databases in terms of methodologic limitations.

According to Calabrese and Baldwin (2001), the first database (20,285 articles screened from three journals) was representative of general studies in experimental toxicology and pharmacology and a valid statistical selection for hormesis screening. However, this claim is quite problematic, given the skewed distribution of dose–response relationships via entry criteria in their Table 2 (Calabrese and Baldwin 2001). The journal *Environmental Pollution* yielded 251 total dose–response relationships from 3,058 articles published over 29 years. More than half [141 (56%) of the total dose responses] appeared during only 3 years (66 in 1987, 50 in 1985, 25 in 1990), with almost half [116 (46%)] appearing in just 2 years, 1985 and 1987. The 141 dose–response relationships appeared in 9 articles, and the 116 in 5 articles.

The journal *Bulletin of Environmental Contamination and Toxicology* produced 206 dose–response relationships from 7,404 screened articles produced over 33 years. Of these, 98 (48%) appeared in just 5 years (17 in 1990, 27 in 1992, 26 in 1995, and 14 in both 1996 and 1997). These are contained in 22 articles. The journal *Life Sciences* yielded 211 dose–response relationships from 9,823 screened articles over 24 years of publication. Of these, 128 (61%) appeared in 44 articles over just 4 years (26 in 1978, 27 in 1985, 33 in 1992, and 42 in 1998).

This clustering of dose–response relationships also appears to show close interyear clustering. For example, the highest tallies for dose responses in *Environmental Pollution*, 50 and 66, occurred 2 years apart, 1985 and 1987, respectively. Markedly clustered distributions raise several questions, such as the presence of serial publications over a relatively short time by a few groups of investigators employing a few experimental protocols, rather than a broad and even distribution of experimentalists and experimental studies. It is not possible from available information published by Calabrese and Baldwin to determine a basis for clustering.

Problems internal to the ad hoc identification and characterization criteria evolved by these researchers affect both the number of articles that meet entry criteria and subsequent evaluation of those articles to confirm hormetic responses. These problems are methodologic and statistical. The criteria have not been independently validated as to reliability, sensitivity, and specificity (Kitchin and Drane 2005; Mushak 2007), thereby weakening the assumption of validated ad hoc search criteria presented earlier.

The large BELLE (Biological Effects of Low Level Exposures) database described by Calabrese and Baldwin (2001, 2003b), among other things, was assembled at the outset to search for and identify the presence of hormetic data. Crump (2001) highlighted some of the special statistical problems associated with data searches and methodologies using criteria established *a priori*. For example, one must correctly control for the false-positive rate in searching databases for a specific form of a phenomenon, for example, hormesis-like data. Crump noted that if one is looking for hormesis-like examples within 100 published dose–response relationships in articles being searched, then the appropriate condition for statistical significance to control for false positives requires that, for the standard level of 0.05, the more stringent *p*-value estimated from the relationship 1 – (1 − *p*)^100^ = 0.05, or *p*= 0.0005, is required.

Calabrese and colleagues have taken general note of Crump’s statistical criticisms, such as replacement of scoring schemes with screening criteria. It is not evident, however, that they have implemented, rationalized, or appropriately validated tests to apply statistical rigidity within screening criteria to minimize type 1 errors through uniform use of more severe statistical tests, as noted by Crump (2001). For example, only one of the multiple tests within the evaluation criteria for ruling in hormetic response and ruling out false-positive frequencies in Tables 4 and 5 and Figure 2 of Calabrese and Baldwin (2001) is the statistically accepted one of statistical significance (*p* ≤ 0.05). The assumption is that other tests show, for example, potential equivalence to statistical significance.

Two components in determining frequency of hormetic responses in the large literature data set of Calabrese and Baldwin (2001) are the hormetic frequency determinations themselves and determinations of the numbers of false positives to permit adjustments to the claimed actual hormetic response rates. The two methodologies link closely conceptually and mathematically, as one cannot determine one without knowing the other. In addition, the determination of frequencies of hormetic effects and frequencies of false positives are both done within the same conceptual and methodologic framework, that is, approaches largely governed by arbitrary and unvalidated entry and evaluation criteria. Consequently, problems with either of these elements for the gross and adjusted frequency assessments attach to the other as well.

The various and largely unvalidated categories of total and hormetic-effect dose responses are set forth in Table 1 and Figures 1 and 2 of Calabrese and Baldwin (2001), and the false-positive estimating types of methodologies are shown in Tables 4–6 of the same paper. They used three types of entry and evaluation methods for estimating the total hormetic frequencies to include for inverse U-shaped hormetic effect curves: statistical significance, data distribution, and alternative quantitative methods, as defined by the authors. The data distribution method, involving absence of overlap in measures of variability, is held to be potentially statistically significant. The alternative quantitative method employed at least three data points where positive deflection from control (a hormetic response in cases of inverted U-shaped curves) was ≥ 110% of control, but it had no statistical basis for validity. Why use only the modest deflection value of 10% over controls? Lastly, the authors presented these methods with such low transparency that the reader must pay particularly close attention in tracking data generation and presentation.

One calculates the unadjusted and adjusted frequencies for the most reliable screening criterion, statistical significance—using Figure 2 and Table 6 of Calabrese and Baldwin (2001)—to be 11% in both cases. By contrast, using the two more arbitrary methods plus that of statistical significance more than triples the figure, to 37% and 35%, respectively (Table 6).

Calabrese and Baldwin (2001) estimated false-positive rates and nonrandomness for hormesis in the data set, that is, employed ratios of the percentage of stimulatory hormetic responses to the percentage inhibitory deflections from controls (false positives). For the case of inverted U-shaped hormetic curves [Figure 1 of Calabrese and Baldwin (2001)], stimulation or positive deflections versus controls below the NOAEL is the numerator and inhibition below the NOAEL similarly applies for the denominator. Calabrese and Baldwin (2001) reported in their abstract that the false-positive rate using their approach is 0.6%, derived from having seven false-positive doses and 1,089 total doses in the portion of the data set with two or more dosing points below the NOAEL.

The above picture becomes much less clear-cut when examining Tables 4 and 5 of Calabrese and Baldwin (2001). Table 4 is the basis for the authors noting the quite low false-positive rate of 0.6% and the associated, relatively robust ratio for hormetic over false-positive effects of 32.5. Table 5, depicting the cases in the overall data set with three or more dosing points ≥ 110% of controls for the evaluation but using hypothesis testing, data distribution, and alternative quantitative methods for the entry criteria, shows a much higher false-positive rate of 22% for the least arbitrary entry criterion of hypothesis testing. The intermediate methodologic approach as to reliability—data distribution—shows a 9% false-positive rate. By contrast, the most arbitrary approach—alternative quantitative—shows a 0% false-positive rate. That is, the least problematic and the most problematic approaches show the highest and lowest false-positive rates, respectively. Last, a mathematical error in Table 5 erroneously presents the sum of positive instances of hormesis as 75 instead of 57.

A critical determinant in how one interprets data of the type described by Calabrese and Baldwin (2001) is the nature of the experimental control populations. Responses in controls are the metrics against which the stimulatory and inhibitory frequencies are compared and determined. This is the case with all three categories of evaluation criteria they described in their article. Furthermore, control responses figure in estimating the relative primacy of hormetic over threshold dose responses as claimed by Calabrese and Baldwin (2003b).

A particular issue with the statistical nature of experimental control populations is that the relative excursions of hormetic responses versus controls are quite modest, and therefore such responses may lie within the collective experimental control population variability when hundreds of different hormetic effects are winnowed from thousands of publications. This is in contrast to the relatively wide differences in occurrence of adverse effects versus controls on dose–response curves above NOAEL values.

Questions arise with respect to this critical role of experimental controls. The level of variability in responses in control groups within very large data sets would be expected to affect the relative reliability of identifying hormetic effects when controls are in the testing protocols within the large screening data sets and affect the frequency of hormetic effects. It is not clear from the available evidence that control group variability can be readily identified and quantified in hormetic characterizations, nor is it clear that hormetic responses are quantitatively in excess of the extent of variability. The analyses of Mayo and Spanos (2008) and Zapponi and Marcello (2006) address the nature of control group responses and their role in hormesis characterization.

Mayo and Spanos (2008) cautioned that adequate attention to deficits in various articles by Calabrese and colleagues, such as absence of statistical significance tests for hormetic responses, described in the critical comments of others, is lacking. They pointed out that hormetic effects are claimed via relatively unsevere tests. They also noted the Calabrese et al. approach, using entry criteria, gives closer scrutiny to cases where such effects occur with a high incidence among controls. Zapponi and Marcello (2006) also addressed idiosyncratic control responses and pointed out a number of situations where claimed hormesis is driven by high-effect incidence in controls and is rarely statistically significant.

Calabrese and Baldwin (2001) claimed that the 20,285-article database revealed 245 hormetic-type responses in 86 articles, 0.42% of the original tally. The total count of 668 dose responses was contained in 195 articles (~ 1%) (Calabrese and Baldwin 2001). The 245 (37%) hormetic responses, however, are derived from a variety of data evaluations, only 74 of which met the minimal accepted scientific criterion of statistical significance (*p* ≤ 0.05) [Figure 2 of Calabrese and Baldwin (2001)]. This statistically significant hormetic dose–response percentage is 11% (74/668) of all dose responses and approximates that found in a study by Davis and Svendsgaard (1994) using data from a broader range of journals, 12–24%.

The second core claim, that hormesis is more common than threshold responses, is rooted in three reports. The first relies on a problematic statistical premise described by Calabrese and Baldwin (2003b). They calculated the ratio of the numbers of dosing points below the NOAEL but above control responses (*A*) to the dosing numbers below the NOAEL and below control response (*B*) for almost 1,800 dosing points contained within 664 dose–response relationships to be 2.5:1, favoring points above control responses (i.e., stimulation). They found this ratio to be significantly different statistically from 1.0. This was taken as evidence of hormesis primacy over threshold responses, as the reverse outcome requires the *A*/*B* ratio be about 1, from a random distribution of dosing points above and below control responses.

However, the data points were not gathered from a purely random sampling within the main database for use in the *A*/B ratio test, but from dose–response relationships meeting the unvalidated, ad hoc entry criteria for data having potential hormetic character and meriting further assessment. The ratio test employed by Calabrese and Baldwin (2003b) considers only deflections above and below controls for isolated dosing points stripped from entry dose responses contained within preselected articles.

A truly random data set for purposes of this particular ratio test should include the additional dose responses with only single sub-NOAEL dosing points in terms of deflections in either direction. These dose responses and publications with them are not statistically confined to the population of dose responses meeting the entry criteria for hormesis screening of two or more individual points below the NOAEL. Randomness in this expected-to-be larger count of individual dosing points (vs. the 1,791 dosing points) combined with the 1,791 dosing points actually employed would reduce the value of any resulting ratio to < 2.5 and potentially to a value not significantly different statistically from 1.0.

A further difficulty, made worse by limited transparency for changes in analysis of data sets over the course of the articles, is that the complementary analysis data reported by Calabrese and Baldwin (2001) showed that 80% of the 1,089 data points below the NOAELs were not different from controls (vs. only about 20% with hormetic character) and therefore consistent with a threshold response model. How the 1,089 dosing points reported by Calabrese and Baldwin (2001) relate 2 years later to the 1,791 data points claimed to clearly show the presence of hormetic over threshold responses (Calabrese and Baldwin 2003b) still awaits explanation.

Last, what is the conceptual and statistical validity of stripping hundreds of individual dosing points from lower numbers of intact dose–response curves representing various substances, numbers of points, end points, experimental and biological systems, etc.? For example, a dose–response curve for substance X with four or five sub-NOAEL dosing points and concurrent controls with a high response rate for the specific end point gives dosing points that would be added to two sub-NO-AEL points on a dose–response curve for substance Y, from a study using controls with a low response rate for that specific end point. On its face, the practice appears to inform the issue of (non)randomness, but the (non)ran-domness of what exactly? Allied to this, why are such analyses not confined to the intact dose–response curve as the statistical unit for testing?

Elliott (2008a) drew attention to some difficulties of ignoring distinctions between isolated dosing points and intact dose–response relationships. One mathematical consequence of the distinction is that if there is a random (i.e., 1:1) distribution of hormetic to threshold responses, the ratio of the corresponding isolated dosing points under certain assumptions would approach 3:1. This ratio can be compared with the ratio estimated by Calabrese and Baldwin (2003b).

Any claim that hormesis is the most common dose–response model requires that it be the major model mathematically and be shown to be so using dose–response data analysis for other candidate dose–response models. One cannot make quantitative (percentage) estimates of hormesis occurrence simply by leaving uncharacterized the residual information remaining after winnowing the hormetic dose responses meeting *a priori* criteria. Second, stating a percentage frequency of one of several options for some quantitative metric (dose–response relationships) assumes one knows the nature of the other dose–response models and their frequencies. Hormesis is clearly not the principal dose–response model in the Calabrese and Baldwin (2001) report, being only 11% (74/668) using the established standard of statistical significance (*p* ≤ 0.05) for the best evidence, or 37% (245/668) of all entry dose–response relationships when using statistical significance plus the various Calabrese and Baldwin (2001) unvalidated evaluation criteria.

What, then, are the dose–response models comprising the balance of the percentages in, first, the Davis and Svendsgaard (1994) data, given that hormesis was sought in all the data sets using several ad hoc criteria but found in only 12–24% of tested cases? The Davis and Svendsgaard database consists of results for noncarcinogens, and the remaining 76–88% of dose responses would be threshold responses. In Calabrese and Baldwin (2001), the 11% value using the statistical significance criterion or their more arbitrary 37% figure suggests that nonhormetic dose–response relationships exist in the majority of cases.

The articles by Calabrese and colleagues describing assessments of hormetic phenomena embedded in data from other journals principally probed the scope of hormesis-like phenomena devoid of frequency estimates. A number of these are noted in Mushak (2007). These later articles presented individual cases of hormetic phenomena, cases not characterized as to their frequency. All one knows is that in the specific cases identified, a hormetic response was claimed.

An important question concerns the scope of the Calabrese and Baldwin frequency figure of 37%, or 11% using the less arbitrary statistical significance test. If the remaining 99.6% of the 20,285 screened articles had contained sufficient dose ranging and met other ad hoc entry and evaluation criteria, what is the likelihood that 37% (or 11%) rather than 0.4% of the 20,285 raw article base would have contained hormesis-revealing dose–response relationships? That is unknown, but Calabrese and Baldwin (2001) leave one to infer 37%.

Calabrese and Baldwin (2003a) also evaluated data from NTP dose-ranging studies and noted these have the advantage, among other things, of using five dosing points per testing. Calabrese and Baldwin (2003a) actually illustrated the high potential for misstatements and contradictions in interpretations and conclusions when using problematic ad hoc methodologies.

Calabrese and Baldwin (2003a) claimed a significant frequency of hormesis across testings for 58 substances examined by the NTP program: 51 substances were said to show hormetic effects, 48% of bioassays involving mice and 14% of bioassays involving rats were said to show hormesis, and hormetic effects were said to be present in 128 of 409 (31%) dose–response relationships. A different picture emerges when looking at the strength of the actual evidence for the 128 hormetic responses. Calabrese and Baldwin (2003a), using a ranking scheme developed earlier, noted that 76% (98/128) of the 128 relationships yielded low evidence of hormesis, 16% (21/128) showed low-to-moderate evidence, 5% (6/128) showed moderate evidence, 1.5% (2/128) showed moderate-to-high evidence, and 0.8% (1/128) showed high evidence.

Of 409 total dose–response relationships, only 0.73% (3/409) showed moderate-to-high or high evidence for hormesis, and only 0.24% (1/409) presented with the most reliable (high) evidence. These frequencies do not provide strong support for the 31% frequency stated elsewhere in the same article or the 37% (245/668) showing the best evidence of hormesis in Calabrese and Baldwin (2001).

The second and third reports asserting the primacy of hormetic over threshold responses are those of Calabrese et al. (2006, 2008). They first reported that their examination of the large NCI yeast response screening database for antitumor drugs demonstrated, when 2,189 antitumor drugs were evaluated via 56,914 dose–response studies (five dosings, 13 yeast strains, replication), that hormetic response patterns were observed about four times more frequently than would be predicted from statistical chance alone. They asserted that their findings call for rejection of the threshold dose–response model in favor of the hormetic one.

It is not known what claimed hormetic responses in yeast systems responding to anti-tumor agents say about hormetic responses in biological systems at large, in human populations, or about the need to substitute hormesis for any other risk assessment dose–response paradigm in public health and regulatory policies. Secondly, use of this database by Calabrese et al. is complicated by the fact that only data summaries, not original data, were preserved by the NCI investigators.

Crump (2007) took note of this gap in original data-handling details in criticizing the Calabrese et al. (2006) results, reporting that at least one of two plausible ways the original NCI investigators could have statistically handled the control well data would eventually yield findings of hormesis-like responses—as a methodologic artifact—without hormesis being present. Calabrese et al. (2007b) rebutted Crump’s claims, insisting that the original testing procedures at statistical issue in the NCI effort were correctly identified on the basis of information from the original investigators and a poll among outside investigators doing this type of research. Calabrese et al. (2007b) further asserted that Crump’s alternative data handling lacked credibility, as it was unlikely that his approach to data handling was used in the NCI studies.

The critical question is which of these two equally plausible approaches for data handling of controls is correct for revealing the truth about whether actual hormetic phenomena are present in data sets generated in these NCI screening protocols, protocols not developed to elucidate hormesis per se. The counter-claim that Crump’s approach for control data handling would probably not have been NCI’s does not appear relevant to this or a second critical question: Why is identification of allegedly robust hormetic responses methodologically unstable in the first place? Identifying robust and stable hormetic effects should be independent of subtle nuances and details such as control data statistical handling. Because two equally plausible alternatives for control well data handling (regardless of what NCI investigators did) reveal two different outcomes regarding hormesis, the question about the validity of using the NCI data to prove robust hormesis commonality remains unresolved.

Calabrese et al. (2008) offered two added claims in further analyses of the NCI data set. First, the percentage differences from controls in overall responses are governed by an overall upshift in observed means versus predicted threshold dose–response means, meaning overall hormetic expressions exist. Second, there are within the suite of tested chemicals a significant number that show strong effects. This study, like some of its predecessors, still raises concerns. First, there is the use of screening entry and other assessment criteria that remain to be validated as to sensitivity and specificity. Low transparency to the methods makes it difficult to judge some of the claims as to consistency with their other work and the objective basis for labeling responses as strong. Furthermore, the matter carried over from the Crump (2007) critique remains unresolved. It is not clear the extent to which the chosen method for dealing with control well responses affects results here versus the Calabrese et al. (2006) analyses. Calabrese et al. (2008) noted design and methodologic differences with Calabrese et al. (2006), but the earlier matter remains unresolved.

An example of ambiguity and inconsistency is the conclusion of Calabrese et al. (2008) that, although certain treatments produced responses < 10% over controls and even responses below controls, these are still hormetic in character. However, the authors did not make clear why such subcontrol (< 100%) responses are not actually an expression of false-positive occurrences, as seen in Calabrese and Baldwin (2001). Furthermore, they held that responses ≤ 80% are inhibitory, further leaving the treatments within the remaining 20% interval from controls uncharacterized.

A second area of ambiguity and low transparency is exactly how the troublesome influence of control variability is sufficiently attenuated in their methodology. See the discussion above and those of Mayo and Spanos (2008) and Zapponi and Marcello (2006) about the critical role of high control group response rates vis-à-vis hormetic frequencies. There are no reliable bounding criteria for objectively categorizing strengths of chemical-specific hormetic responses in Calabrese et al. (2008). In semantic terms, we have chemicals that produce differences in the end points claimed for hormesis but that may not translate to variable strength in hormetic responses.

Finally, this second effort leaves intact the major concern about relevance of yeast strain results in Calabrese et al. (2006) to potential hormetic effects in humans and other higher biological systems. At the end of the second analytical and interpretive day, we are still limited to yeast systems.

## The Language of Hormesis Characterization

The most recent incarnation of hormesis has been accompanied by a tailored vocabulary for the topic coupled with problems in language precision, yielding ambiguous and potentially misleading terminology. Language matters. Language especially matters with evolving and/or controversial scientific topics where communicating and interpreting new findings must be done free of ambiguity. Parts of this customized vocabulary are not only questionable uses of settled meanings within the broader language but also serve to inflate such characteristics as the claimed scope and frequency of hormetic phenomena in nature or published literature, and conflate hormesis in importance with existing pillars of risk assessment and toxicology and with the universality of other biological phenomena.

The first language challenge is definition. Repeated redefinitions of hormesis have had mixed effect in systematically and convincingly answering what hormesis is, why it exists, and why it is important. It is not clear that any final and clear definition exists for hormesis. Calabrese and Baldwin (2002) attempted a fairly comprehensive definition of the nature and underlying biological purpose of hormesis in nature, settling on hormesis as an adaptive response.

More recently, Calabrese and a large number of coauthors attempted to present the rationales for an even more extensive definition (Calabrese et al. 2007a). Three expressions of the term were proffered, two centering on adaptive or conditioning phenomena in response to biological stress, and the third encapsulating the conventionally understood form of hormesis. In this scheme, conditioning/adapting stresses and post-exposure conditioning responses are allied with the conventional phenomenon of hormesis, with the latter not evincing conditioning or adapting dose responses. Where there was an interim notion, for example, of just “chemical hormesis,” there are now two additional terms for responses to chemicals: “chemical conditioning hormesis” and “chemical postexposure conditioning hormesis.”

The epistemologic difficulty with this attempted broader taxonomy by Calabrese et al. (2007a) is that it uproots the assumption that chemical hormesis is a discrete phenomenon having a defensible definition that is amenable to a discrete quantitative characterization within reliable criteria while governed by a discrete overarching mechanism or mechanisms, and has a discrete function in nature that would better abet acceptance of a role in health and regulatory policy.

Hormesis as a specific term and concept, in terms of biological functions and mechanistic underpinnings, cannot be three different biological phenomena simultaneously. These floating expressions need to give way to discrete, differentiating labels and a reassembling of current evaluatory approaches and databases to adequately validate each separately.

The use of various terms for generalizability by hormesis advocates differs markedly from the broader definitions and uses of these terms (Mushak 2007). Cook and Calabrese (2006) referred to hormetic responses being “ubiquitous” in nature, but that definition (Merriam-Webster 2002) requires hormesis to be everywhere at all times (i.e., with 100% frequency of occurrence and with 100% frequency at any and all times within a given testing or testing series).

Calabrese et al. (1999) held hormesis to be a “highly generalizable and reproducible” phenomenon. “Generalizability,” although less semantically stringent than “ubiquity,” presupposes that hormesis, being highly generalizable, occurs in nature or in the literature with at least a more-likely-than-not occurrence (i.e., > 50%) in screened and evaluated literature. The evidence does not support this. Data from two sets of investigators indicate absence of a 100% or even > 50% frequency of occurrence in published literature (Calabrese and Baldwin 2001; Davis and Svendsgaard 1994). Nonetheless, the term is intractably ambiguous. Use of the term “reproducible” (Calabrese et al. 1999) as a characteristic of hormesis is equally ambiguous; these authors took the term to mean examples of hormetic responses for similar substances or classes across studies, whatever the frequency. Conventional use of the term is that different investigators get basically the same results under the same set of experimental conditions.

One troubling element of this customized vocabulary is the uncoupling or muddling of the link between frequency of occurrence and frequency as a critical determinant of generalizability. Frequency of hormetic examples is treated separately by the UMass-Amherst group (Calabrese and Baldwin 2001) and is confined to analysis of a discrete database by Calabrese and colleagues. Frequency estimations for hormesis do not appear as a core component of analysis of all the databases examined by Calabrese and colleagues. Generalizability for hormesis is apparently established here if one sees hormetic examples, regardless of overall frequency. This applies to the numerous examples summarized in the special issues of *Critical Reviews in Toxicology* as cited by Mushak (2007).

Finally, proponents appear to be inconsistent in applying the language for hormesis. Calabrese and Baldwin (2002) noted that hormesis defined as “direct stimulation” is modest stimulation, with too much stimulation depicting some other phenomenon. This stance contradicts the basic definition of hormetic behavior, that is, the phenomenon is biphasic. The more stimulatory its expression, the more apparent the presence of hormesis. Their stance, furthermore, does not void the alternative possibility that modest deflections from controls are statistical artifacts and that strong stimulation is actually hormesis.

## Conclusions

### What has been accomplished with hormesis over the last 10–15 years?

Hormesis as term and concept is now less obscure and neglected than in past decades. A significant volume of information has appeared, much from the hormesis group at UMass-Amherst (Calabrese and colleagues). Although both chemical and radiation hormesis have been of interest to proponents, the former has been most heavily evaluated. Collateral awareness has been raised in various scientific and health policy quarters about the central role of quantitative toxicology in the toxicologic and related health sciences via the dose–response relationship. Recent advocacy efforts by hormesis proponents also illustrate that in any advocacy there is some measure of devil’s advocacy. That is, evaluation of hormetic response models forces equal scrutiny of other dose–response models, whatever the technical merits, limitations, or plausibility of hormesis.

### What has not been accomplished with hormesis in the last 10–15 years?

Hormesis as a term and concept remains riddled with gaps, serious and valid questions about the science, and skepticism in scientific quarters over the relevance of hormetic phenomena to human health risk assessment and various regulatory policies. To date, hormesis has not been subjected to systematic and arms-length collective scrutiny by scientific organizations such as the National Academy of Sciences/National Research Council or other recognized entities.

Furthermore, no public agency has adopted hormesis as part of risk assessment or regulatory policy or expressed any inclination to do so. Reasons for this inaction likely include lingering skepticism, the topic’s relative immaturity, the nature of how activity on the topic over the last 10–15 years has been pursued, and even how one would do a hormetic risk assessment.

The type and number of efforts by such proponents as the UMass-Amherst hormesis group have significantly formed and advanced the current status of hormesis, and it is unlikely that hormesis would be at its present stage without that involvement. Still open is the question of whether the efforts of the proponents simply accelerated and forced streamlining of the scientific gatekeeping process for hormesis and any potential roles in health, regulation, or other sectors versus taking the field in specific directions.

The specific methodologies and strategies employed by these workers in accelerating the study and promotion of hormesis have taken development of the topic in specific developmental directions. The issue of the extent to which such progress has engendered any ethical dimensions has been addressed. Elliott (2008b) provided a broad analysis of ethical matters in summarizing a group of articles on the topic included in the August 2008 issue of *Human and Experimental Toxicology*.

Broad scientific acceptance of new or controversial biological phenomena seems a plodding and multidirectional process, and one that might well invite impatience. This impatience would be amplified in those who accept that hormesis is basic biology and toxicology and also has something important to say about biological phenomena in general and dose–response toxicology and pharmacology in particular. Calabrese (2002) stated that previous incarnations of hormesis failed to be accepted because of “a complete lack of strong leadership to advocate its acceptance in the right circles.”

Rooted in any seemingly plodding pace of scientific oversight, however, are many mechanisms for auditing the potential survival of junk science, providing objective expert consensus through arms-length means, and providing wide scientific acceptance because of the efforts of many, not one or a very few.

### Where to for hormesis?

Major advances in chemical hormesis beyond empirical curiosity will require convincing evidence that the phenomenon is relevant to higher orders of biological complexity, especially for dose–response relationships in human populations. Such advances would have to precede considering chemical hormetic phenomena in human risk assessment and regulatory options. Biological relevance of chemical hormesis to human populations is hobbled by the same comparative empirical conundrum affecting other areas of biology and toxicology, especially given its provisional status. The simpler the study system, the more easily characterizable, but the less its relevance to humans; the more biologically complex and closer the study system as a human response surrogate, the less easy the characterization, but the more confidence about relevance to humans. From the UMass-Amherst efforts, we have a good number of articles on relatively simple biological systems but little hard biological evidence of the nature and extent to which hormesis operates in humans and human populatons.

## References

[b1-ehp-117-1333] Calabrese EJ (2001). The future of hormesis: where do we go from here?. Crit Rev Toxicol.

[b2-ehp-117-1333] Calabrese EJ (2002). Hormesis: changing view of the dose-response, a personal account of the history and current status. Mutat Res.

[b3-ehp-117-1333] Calabrese EJ (2008). Hormesis: why is it important to toxicology and toxicologists. Environ Toxicol Chem.

[b4-ehp-117-1333] Calabrese EJ, Bachmann KA, Bailer AJ, Bolger PM, Borak J, Cai L (2007a). Biological stress response terminology: integrating the concepts of adaptive response and preconditioning stress within a hormetic dose-response framework. Toxicol Appl Pharmacol.

[b5-ehp-117-1333] Calabrese EJ, Baldwin LA (2001). The frequency of U-shaped dose responses in the toxicological literature. Toxicol Sci.

[b6-ehp-117-1333] Calabrese EJ, Baldwin LA (2002). Defining hormesis. Hum Exp Toxicol.

[b7-ehp-117-1333] Calabrese EJ, Baldwin LA (2003a). Hormesis at the National Toxicology Program (NTP): evidence of hormetic dose responses in NTP dose-range studies. Nonlinearity Biol Toxicol Med.

[b8-ehp-117-1333] Calabrese EJ, Baldwin LA (2003b). The hormetic dose-response model is more common than the threshold model in toxicology. Toxicol Sci.

[b9-ehp-117-1333] Calabrese EJ, Baldwin LA, Holland CD (1999). Hormesis: a highly generalizable and reproducible phenomenon with important implications for risk assessment. Risk Anal.

[b10-ehp-117-1333] Calabrese EJ, Blain R (2005). The occurrence of hormetic dose responses in the toxicological literature, the hormesis database: an overview. Toxicol Appl Pharmacol.

[b11-ehp-117-1333] Calabrese EJ, Stanek EJ, Nascarella MA, Hoffmann GR (2008). Hormesis predicts low-dose responses better than threshold models. Int J Toxicol.

[b12-ehp-117-1333] Calabrese EJ, Staudenmayer JW, Stanek EJ, Hoffmann GR (2006). Hormesis outperforms threshold model in NCI anti-tumor drug screening data. Toxicol Sci.

[b13-ehp-117-1333] Calabrese EJ, Staudenmayer JW, Stanek EJ, Hoffmann GR (2007b). Hormesis and high throughput studies: Crump’s analysis lacks credibility [Letter]. Toxicol Sci.

[b14-ehp-117-1333] Cook R, Calabrese EJ (2006). The importance of hormesis to public health. Environ Health Perspect.

[b15-ehp-117-1333] Crump K (2001). Evaluating the evidence for hormesis: a statistical perspective. Crit Rev Toxicol.

[b16-ehp-117-1333] Crump KS (2007). Limitations in the National Cancer Institute antitumor drug screening database for evaluating hormesis [Letter]. Toxicol Sci.

[b17-ehp-117-1333] Davis JM, Svendsgaard DJ, Calabrese EJ (1994). Nonmonotonic dose-response relationships in toxicological studies. Biological Effects of Low Level Exposures: Dose-Response Relationships.

[b18-ehp-117-1333] Elliott KC (2008a). A case for deliberation in response to hormesis research. Hum Exp Toxicol.

[b19-ehp-117-1333] Elliott KC (2008b). Hormesis, ethics and public policy: an overview. Hum Exp Toxicol.

[b20-ehp-117-1333] Kitchin KT, Drane JW (2005). A critique of the use of hormesis in risk assessment. Hum Exp Toxicol.

[b21-ehp-117-1333] Mayo DC, Spanos A (2008). Risks to health and risks to science: the need for a responsible “bioevidential” scrutiny. Hum Exp Toxicol.

[b22-ehp-117-1333] Merriam-Webster, Inc. (2002). Webster’s Third New International Dictionary, Unabridged.

[b23-ehp-117-1333] Mushak P (2007). Hormesis and its place in nonmonotonic dose–response relationships: some scientific reality checks. Environ Health Perspect.

[b24-ehp-117-1333] Zapponi GA, Marcello L (2006). Low-dose risk, hormesis, analogical and logical thinking. Ann NY Acad Sci.

